# Multi-task deep learning for automatic image segmentation and treatment response assessment in metastatic ovarian cancer

**DOI:** 10.1007/s11548-025-03484-0

**Published:** 2025-09-03

**Authors:** Bevis Drury, Inês P. Machado, Zeyu Gao, Thomas Buddenkotte, Golnar Mahani, Gabriel Funingana, Marika Reinius, Cathal McCague, Ramona Woitek, Anju Sahdev, Evis Sala, James D. Brenton, Mireia Crispin-Ortuzar

**Affiliations:** 1 Department of Physics, University of Cambridge, Cambridge, United Kingdom; 2 Department of Oncology, University of Cambridge, Cambridge, United Kingdom; 3 Early Cancer Institute, University of Cambridge, Cambridge, United Kingdom; 4 jung diagnostics GmbH, Hamburg, Germany; 5 Cancer Research UK Cambridge Institute, Cambridge, United Kingdom; 6 Cancer Research UK Cambridge Centre, Cambridge, United Kingdom; 7 Cambridge University Hospitals NHS Foundation Trust, Cambridge, United Kingdom; 8 Research Center for Medical Image Analysis and AI, Danube University, Krems an der Donau, Austria; 9 Department of Radiology, Barts Health NHS Trust, London, United Kingdom; 10 Department of Radiologic Sciences, Università Cattolica del Sacro Cuore, Milan, Italy

**Keywords:** Multi-task deep learning, Image segmentation, Treatment response assessment, Metastatic ovarian cancer

## Abstract

**Purpose:**

High-grade serous ovarian carcinoma (HGSOC) is characterised by significant spatial and temporal heterogeneity, often presenting at an advanced metastatic stage. One of the most common treatment approaches involves neoadjuvant chemotherapy (NACT), followed by surgery. However, the multi-scale complexity of HGSOC poses a major challenge in evaluating response to NACT.

**Methods:**

Here, we present a multi-task deep learning approach that facilitates simultaneous segmentation of pelvic/ovarian and omental lesions in contrast-enhanced computerised tomography (CE-CT) scans, as well as treatment response assessment in metastatic ovarian cancer. The model combines multi-scale feature representations from two identical U-Net architectures, allowing for an in-depth comparison of CE-CT scans acquired before and after treatment. The network was trained using 198 CE-CT images of 99 ovarian cancer patients for predicting segmentation masks and evaluating treatment response.

**Results:**

It achieves an AUC of 0.78 (95% CI [0.70–0.91]) in an independent cohort of 98 scans of 49 ovarian cancer patients from a different institution. In addition to the classification performance, the segmentation Dice scores are only slightly lower than the current state-of-the-art for HGSOC segmentation.

**Conclusion:**

This work is the first to demonstrate the feasibility of a multi-task deep learning approach in assessing chemotherapy-induced tumour changes across the main disease burden of patients with complex multi-site HGSOC, which could be used for treatment response evaluation and disease monitoring.

## Introduction

Despite significant progress in ovarian cancer treatment, it remains one of the leading causes of gynaecological cancer-related deaths among women [1]. High-grade serous ovarian carcinoma (HGSOC) is the most prevalent type and is characterised by considerable heterogeneity, often presenting as an advanced multi-site metastatic disease [2]. Neoadjuvant chemotherapy (NACT) followed by delayed primary surgery is increasingly becoming the standard treatment approach for advanced HGSOC [3]. However, the effectiveness of NACT is complicated by the heterogeneous responses of HGSOC, where some lesions shrink or disappear while others remain stable, grow, or even new lesions develop during treatment [4]. HGSOC patients in an advanced metastatic stage often face disease spread across multiple anatomical locations [5], potentially divided into dozens of separate lesions. This extensive and fragmented nature of the disease makes automated segmentation particularly challenging. Additionally, the response of these individual lesions to treatment is known to influence disease progression and provides valuable predictive insights regarding the effectiveness of delayed primary surgery and patient survival [6]. However, there are currently no automated algorithms to consistently assess the radiological response across all these lesions in metastatic ovarian cancer.

In current clinical practice, tumour response evaluation typically relies on two-dimensional measurements to estimate changes in tumour size from serial contrast-enhanced computed tomography (CE-CT) scans. Although Response Evaluation Criteria in Solid Tumours (RECIST 1.1) is the current clinical guideline to assess the size change of solid tumours after therapeutic treatment, it is based on measurements of lesion diameters and, therefore, is not able to capture the three-dimensional complexity of tumours. Additionally, radiologists have to choose which target lesions to track and measure, which adds subjectivity to the clinical evaluation process. In advanced-stage ovarian cancer with multi-site peritoneal disease, the detailed segmentation and annotation of a single scan is very time-consuming and is often performed only for research purposes, thereby omitting potentially relevant information from clinical decision-making.

**Fig. 1:**
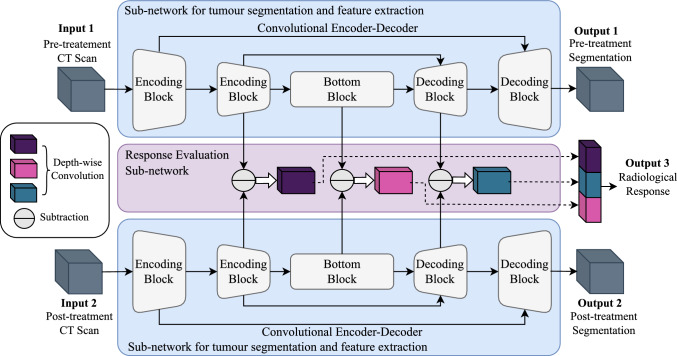
Network model for simultaneous tumour segmentation and response prediction. The network consists of two main components: (1) a convolutional encoding/decoding subnetwork for feature extraction and tumour segmentation, and (2) a multi-stream Siamese subnetwork for response evaluation. The feature extraction and segmentation subnetwork consists of two identical 3D U-Net with shared parameters. The response evaluation subnetwork combines the extracted image features from three different network layers via depth-wise convolution

Our group has recently proposed OvSeg, the first deep learning-based segmentation network for metastatic ovarian cancer [7]. The model achieved a Dice Similarity Coefficient (DSC) of $0.71\pm 0.20$ for pelvic/ovarian and $0.61\pm 0.24$ for omental lesions. However, there is still an unmet need for deep learning methods that effectively combine tumour segmentation and response assessment in metastatic ovarian cancer, which are traditionally treated as separate problems in medical image analysis. Integrating these interconnected tasks into a unified model has been shown to improve prediction performance in solid tumours [8], but this approach has not yet been explored for advanced, multi-site metastatic diseases such as HGSOC. Here, we propose a multi-task deep learning approach that facilitates simultaneous segmentation of pelvic/ovarian and omental tumour lesions in CE-CT scans, as well as treatment response assessment in metastatic ovarian cancer. This work is the first to demonstrate that the integration of the two tasks in one network coupled with the incorporation of change information in longitudinal images improves accuracy for response prediction in HGSOC.

## Methods

### Network model

The network architecture consists of two subnetworks: one for feature extraction and tumour segmentation and one for response assessment, as illustrated in Fig. 1. The segmentation subnetwork is a 3D U-Net [9] containing a contracting path, an expansive path, and skip connections between the corresponding layers. The subnetwork for response assessment combines the extracted image features via depth-wise convolution from three distinct network layers: (1) the intermediate layer in the contracting path, (2) the bottom layer of the U-Net, and (3) the intermediate layer of the combination module at the end of the U-Net as described in a previous work [8]. By doing so, we ensure that both shallow and deep feature representations obtained by the segmentation network can be fully integrated and applied to the response evaluation task. The response prediction subnetwork computes the difference between the pre- and post-treatment feature maps by voxel-wise subtraction. These difference maps are processed by a depth-wise convolutional layer with 32 output channels. The global average of each channel is then concatenated and passed through a linear layer to produce a single response prediction output. We use a Siamese network architecture consisting of two identical 3D U-Nets with shared parameters: one for the pre-treatment scan and one for the post-treatment scan. Both U-Nets segment the lesions independently. The extracted feature maps are then passed to a response prediction subnetwork, which computes their difference to predict treatment response. Sharing weights between the two U-Nets reduces model complexity and encourages generalisable feature learning across time points, particularly beneficial in settings with limited training data. OvSeg performs state-of-the-art segmentation using a U-Net cascade consisting of a low-resolution network whose output is upsampled and passed to a full-resolution network. In contrast, our approach employs a single-stage segmentation process using a 3D U-Net with randomly initialised weights and achieves comparable performance.

### Network training

**Architecture.** At each level in the contracting path, a 3 $\times$ 3 $\times$ 3 convolution with stride 2 is performed to double the number of feature maps while reducing the resolution. This is followed by a context module or pre-activation residual block with two 3 $\times$ 3 $\times$ 3 convolutional layers and a dropout layer in between. Each level of the expansive path begins with a localisation module consisting of a 3 $\times$ 3 $\times$ 3 convolution, which upsamples the feature maps to a higher resolution. In this study, the batch size was set to 2. **Loss function.** The model was trained using a weighted sum of the segmentation and response prediction losses. The segmentation loss was computed as a weighted combination of Dice and cross-entropy losses, applied to downsampled segmentations from each decoder layer of the U-Net following the strategy adopted in [7]. The cross-entropy loss function was used for response prediction. Equations (1) and (2) show the Dice and cross-entropy loss functions, respectively, where *C* is the number of segmentation classes, *y* and ${\hat{y}}$ are the softmax outputs from the predicted and ground-truth segmentations, respectively, and $\epsilon$ is a small value to avoid divergences. In our training, $C=3$, as we calculate the segmentation loss across three classes: background, omental, and pelvic/ovarian lesions. The total loss was a weighted sum of segmentation ${\mathcal {L}}_{seg}$ and response prediction losses ${\mathcal {L}}_{class}$, shown in equation (3), where we took $\lambda _1$ and $\lambda _2$ to be 1 and 0.2 as suggested in [8].

1 $$\begin{aligned} {\mathcal {L}}_{DSC}= &   1- \frac{1}{C} \sum _{c=1}^C \frac{ 2 \sum _i {\hat{y}}_{i, c} y_{i, c} + \epsilon }{ \sum _i ({\hat{y}}_{i, c} + y_{i, c}) + \epsilon } \end{aligned}$$

2 $$\begin{aligned} {\mathcal {L}}_{CE}= &   -\frac{1}{N} \sum _{n=1}^N w_{{\hat{y}}_n} \log \frac{\exp \left( y_{n, {\hat{y}}_n}\right) }{\sum _{c=1}^C \exp \left( y_{n, c}\right) } \end{aligned}$$

3 $$\begin{aligned} {\mathcal {L}}= &   \lambda _1 {\mathcal {L}}_{class} + \lambda _2 {\mathcal {L}}_{seg} \end{aligned}$$

**Training strategies.** We employed established techniques to minimise the risk of overfitting, including data augmentation, instance normalisation, early stopping, and learning rate decay during the training process. The learning rate schedule used a linear warm-up over the first $4\%$ of the training steps, followed by cosine decay. The maximum learning rate was set to $5 \times 10^{-5}$. Training was stopped early at approximately 120,000 iterations based on response prediction performance. To improve generalisation, we applied various augmentation strategies to each training batch. Baseline (BL) and follow-up (FU) scans underwent independent grey value transformations, while sharing the same affine transformation matrix to ensure co-alignment. Grey value transformations included shifting, scaling, Gaussian noise addition, Gaussian smoothing, and contrast adjustment. Each transformation had an independent application probability. Spatial transformations including flipping, scaling, shearing, and rotation along each axis were applied using a random affine matrix, again with individual probabilities. These were combined into a single transformation for computational efficiency. Image volumes were interpolated using bilinear interpolation, while segmentation masks were interpolated using nearest-neighbour interpolation to retain discrete class labels. **High-performance computing.** Training was performed on the University of Birmingham Baskerville HPC cluster using NVIDIA A100 40GB GPUs.

## Experiments

### Patient cohorts

**Fig. 2:**
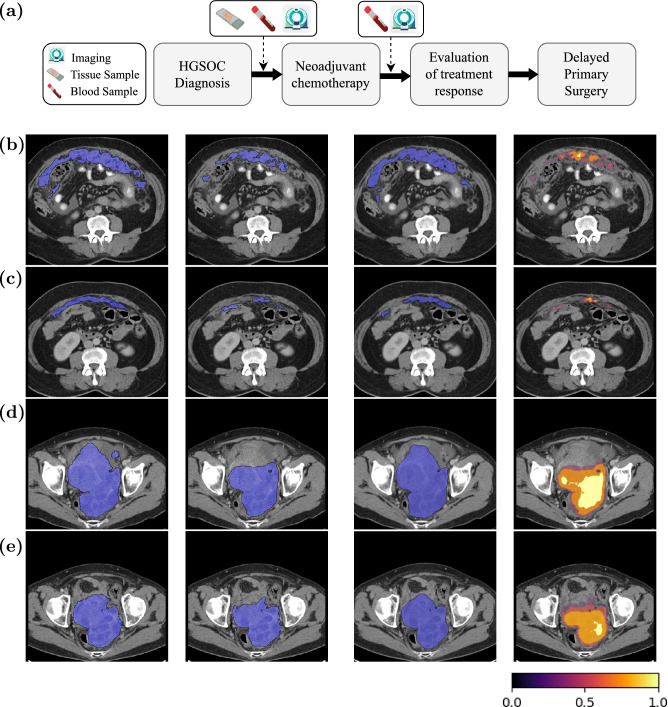
**a** Overview of the treatment timeline and characteristics for an advanced-stage high-grade serous carcinoma. Initial staging and diagnosis were based on CE-CT imaging and biopsy. **b–e** Example axial slices illustrating: from left to right, ground-truth segmentations, our model’s predicted segmentations, OvSeg’s predicted segmentations, and our model’s Grad-CAM maps. **b** and **c** correspond to pre- and post-treatment scans, respectively, from a patient who responded to chemotherapy. **d** and **e** show pre- and post-treatment scans from a patient who did not respond to chemotherapy

This work includes two prospective observational studies from Addenbrooke’s Hospital (‘OV04’, n=99) and the Barts Health NHS Trust (‘Barts’, n=49). All patients had a confirmed histopathological diagnosis of HGSOC, and underwent neoadjuvant chemotherapy before delayed primary surgery. The OV04 patients were treated at Cambridge University Hospitals NHS Foundation Trust and were recruited into a clinical observational study approved by the local research ethics committee (REC reference number: 08/H0306/61). All patients within the Barts dataset were treated at Barts Health NHS Trust between 2009 and 2018 and recruited into a prospective clinical study approved by the local research ethics committee (IRAS reference numbers: 243824). The OV04 dataset was used for the exploratory analyses and to train the deep learning model. The Barts data were used as an independent, external testing dataset. Figure 2 (a) shows an overview of the treatment schedule of advanced-stage high-grade serous carcinoma.

### Image acquisition and labelling

Clinical CE-CT scans covering the abdomen and pelvis were acquired. The radiological response is measured based on the RECIST 1.1 criteria and is categorised into four groups: complete response (CR), partial response (PR), stable disease (SD), or progressive disease (PD). They were then combined into responders and non-responders to address class imbalance. On CE-CT axial images reconstructed with a slice thickness of 5 mm, all cancer lesions were segmented by a board-certified radiologist with ten years of experience in clinical imaging using Microsoft Radiomics (project InnerEye; Microsoft, Redmond, WA, USA). Figure 2 (b) illustrates examples of CE-CT scans demonstrating the metastatic nature of the disease, identifying 16 sites of disease burden with varying numbers of lesions and highlighting heterogeneity in lesion response.

### Image processing

Each scan was attenuation-clipped outside [-150, 250] HU, normalised to the range (0, 1), and resampled to a voxel spacing of $5mm\times 0.8mm\times 0.8mm$. Pairs of pre- and post-treatment scans were spatially co-registered using rigid registration [10], considering the fixed anatomy of the pelvis.

### Evaluation of model performance

We evaluated the accuracy of response prediction using receiver operating characteristic (ROC) analysis. The area under the ROC curve (AUC), sensitivity and specificity, and positive and negative predictive values were calculated. The optimal cut-off point was determined by maximising Youden’s index on the ROC curve. Next, we sought to understand which areas of the image contributed to the network’s output using Gradient-weighted Class Activation Maps (Grad-CAM).

## Results and discussion

**Model performance for tumour segmentation.** The predicted segmentations were compared against the ground-truth segmentations using the DSC score. The model achieved a Dice Similarity Coefficient (DSC) of $0.56\pm 0.12$ for pelvic/ovarian and $0.45\pm 0.67$ for omental lesions. Figure 2 (b–e) shows examples of ground-truth and predicted segmentations and Grad-CAM maps for pelvic/ovarian and omental lesions. The high-intensity visuals reflect the area of interest to our model at the time of prediction. **Model performance for response prediction.** The AUC for response prediction was as 0.78 (95% CI [0.70–0.91]). The maximum weighted average $F_1$ score was 0.72 for a classification threshold range of (0.85, 0.95). The sample weighted average precision, recall, and $F_1$ score were 0.78, 0.76, and 0.72, respectively. The sensitivity and specificity were 0.88 and 0.64, respectively, indicating that the model correctly classifies 88% of responders and 64% of non-responders.

The observed DSC scores were slightly lower than those achieved by OvSeg: 0.56 vs. 0.71 for pelvic/ovarian lesions and 0.45 vs. 0.61 for omental lesions. This performance gap is likely due to the use of only half the training data available to OvSeg and the prioritisation of response prediction in our model, achieved by assigning a higher weight to the response prediction loss relative to the segmentation loss. We anticipate improving these results by incorporating additional training data. Patients with a complete or partial response on CE-CT were considered as responders, and those with stable or progressive disease were considered as non-responders. The model shows greater ability in detecting responders compared to non-responders. The higher sensitivity suggests that the dramatic and observable tumour shrinkage in responders makes them easier to identify accurately. In contrast, the lower specificity implies challenges in distinguishing non-responders, as their tumours may show slight changes that could be misinterpreted as a response.

Our study is a proof-of-principle of using multi-task deep learning to automatically segment metastatic disease and predict the response of HGSOC patients to NACT. However, this study has some limitations and future work is needed to validate the model in larger and more diverse cohorts. Future work includes: (1) investigating alternative model architectures and conducting an ablation study to understand the contribution of each network component, which could provide insights into optimising segmentation and prediction accuracy, (2) expanding segmentation to include additional metastatic sites beyond the omental and pelvic/ovarian regions, and (3) exploring ways to incorporate multimodal data such as clinical blood biomarkers to enhance model robustness. Further clinical developments could have a significant impact as a stratification tool in clinical settings as for example avoiding delays in surgery for patients who are unlikely to respond to chemotherapy.

## Conclusion

Assessing treatment response after NACT is crucial for effectively planning the next stages of the ovarian cancer patient’s clinical pathway. This work proposes a multi-task deep learning model to automatically predict tumour segmentations and evaluate treatment response for HGSOC patients, showing some encouraging preliminary results. With accurate predictions, this model has the potential to improve the reproducibility of response predictions, offering a valuable tool for decision-making.
